# Visual Information Fusion through Bayesian Inference for Adaptive Probability-Oriented Feature Matching

**DOI:** 10.3390/s18072041

**Published:** 2018-06-26

**Authors:** David Valiente, Luis Payá, Luis M. Jiménez, Jose M. Sebastián, Óscar Reinoso

**Affiliations:** 1 Department of Systems Engineering and Automation, Miguel Hernández University, Av. de la Universidad s/n. Ed. Innova., 03202 Elche (Alicante), Spain; lpaya@umh.es (L.P.); luis.jimenez@umh.es (L.M.J.); o.reinoso@umh.es (Ó.R.); 2 Centre for Automation and Robotics (CAR), UPM-CSIC, Technical University of Madrid, C/ José Gutiérrez Abascal, 2, 28006 Madrid, Spain; jsebas@etsii.upm.es

**Keywords:** omnidirectional imaging, visual localization, catadioptric sensor, visual information fusion

## Abstract

This work presents a visual information fusion approach for robust probability-oriented feature matching. It is sustained by omnidirectional imaging, and it is tested in a visual localization framework, in mobile robotics. General visual localization methods have been extensively studied and optimized in terms of performance. However, one of the main threats that jeopardizes the final estimation is the presence of outliers. In this paper, we present several contributions to deal with that issue. First, 3D information data, associated with SURF (Speeded-Up Robust Feature) points detected on the images, is inferred under the Bayesian framework established by Gaussian processes (GPs). Such information represents a probability distribution for the feature points’ existence, which is successively fused and updated throughout the robot’s poses. Secondly, this distribution can be properly sampled and projected onto the next 2D image frame in t+1, by means of a filter-motion prediction. This strategy permits obtaining relevant areas in the image reference system, from which probable matches could be detected, in terms of the accumulated probability of feature existence. This approach entails an adaptive probability-oriented matching search, which accounts for significant areas of the image, but it also considers unseen parts of the scene, thanks to an internal modulation of the probability distribution domain, computed in terms of the current uncertainty of the system. The main outcomes confirm a robust feature matching, which permits producing consistent localization estimates, aided by the odometer’s prior to estimate the scale factor. Publicly available datasets have been used to validate the design and operation of the approach. Moreover, the proposal has been compared, firstly with a standard feature matching and secondly with a localization method, based on an inverse depth parametrization. The results confirm the validity of the approach in terms of feature matching, localization accuracy, and time consumption.

## 1. Introduction

There is a growing tendency for the use of visual sensors, to the detriment of the range sensory data approaches [1,2]. Visual sensors, which are essentially represented by digital cameras, have contributed with valuable advantages to the state of the art [3,4], such as the ability to acquire large amounts of information with only one snapshot. They have become a robust alternative to former sensors, and thus they have been extensively integrated in the framework of localization, in mobile robotics. In particular, they can perform as the main sensor [5,6,7], where no other sensory data are used, and can assist as a secondary sensor [8,9] where the main sensor is unable to produce measures, for instance under GPS (Global Positioning System)-denied circumstances, in unmanned vehicle applications [10].

We have concentrated on catadioptric systems, such as omnidirectional cameras, due to their ability to capture large scenes and their wider field of view, in comparison to planar cameras. Different omnidirectional visual approaches have been proposed. They can be categorized according to the sort of method that processes the visual content of a scene. First, some approaches make use of specific visual points in an image (local feature methods or visual landmark methods) [7,11]. Additionally, a more recent research line has come up with global appearance or holistic methods, relying on the processing of the image as a whole [12,13]. Despite the fact that these recent advances have evidenced a pronounced growth in the efficiency, we have opted for using local feature methods since they have been vastly accepted and tested in terms of performance [14,15], accuracy [7,16], and robustness [17,18].

Nevertheless, both processing methods are required to associate visual data correctly, regardless of the final application, they are intended for. This is a non-trivial aspect that implies an important challenge, which sometimes results in a general issue in many mobile robotics applications [19,20,21]. In this sense, visual features matching [22,23] is one of the most extended techniques in order to describe and associate visual features from one image to another, by comparing certain pixel description metric. Unfortunately, the final estimation typically reflects the harmful effect of false positives in the data association, denoted as outliers. A considerable amount of research in this area has been conducted [24,25,26,27,28]. Nevertheless, the rejection methods normally need substantial computational efforts and external requirements [29,30], beyond the specifications of the target system application.

In this work, we propose an adaptive matching approach, which takes the most of the same visual data input used by our former localization approach [31], which is aided by the odometer’s prior in order to estimate the scale factor. To that end, the visual data are fused at every motion step of the vehicle by means of a Bayesian technique, namely Gaussian processes (GPs) [32]. Such a scheme permits inferring a model of the environment that accounts for the probability of feature existence in the 3D global reference system. In this manner, obtaining a reliable probability distribution permits identifying relevant areas from which some visual features might be detected, in terms of probability of existence. This idea inherits the foundations from exploration models [33], which are aimed at discovering new areas in the environment, and fusing the new information into the estimated models.

The design of these contributions seeks a more realistic approach, with the objective of obtaining robust results in challenging environments, ensuring computational efficiency. Synthesizing, the main differences and contributions of this paper, in contrast to the previous work [31], are as follows:

- The probability framework considers the 3D global reference system, instead of a 2D image frame representation.
- A 3D probability distribution is computed and projected onto the next image, associated to the next pose of the robot, by means of a filter-motion prediction stage. Such probability projection represents relevant areas on the image, where matching detection is more probable.
- The matching process is performed in a single batch, using the entire set of feature points associated with the probability areas projected on the image, instead of a multi-scaled matching, computed feature by feature.
- The information metric permits modulating the probability values for the probability areas, instead of simply representing a set of less precise coefficients for weighting the former multi-scaled matching.

Finally, since this work pursues the achievement of quantitative benefits, a specific application of visual localization has been considered. In this context, different publicly available real datasets have been used in the experimental section, in order to confirm the validity of the approach, and to evaluate its final performance when producing robust and adaptive probability-oriented visual feature matching. Furthermore, extended comparison results have been generated to reinforce and highlight the improvements of the approach, after embedding the implementation into a visual localization application.

The remainder of this paper is structured as follows. First, a general overview to the omnidirectional vision system is provided in Section 2; Section 3 describes the design of the localization model, which relies on the adaption of the epipolar constraint to the omnidirectional geometry of the vision system [31]; the main contributions, regarding the design of the probability distribution of feature points’ existence, are presented in Section 4; Section 5 comprises the experiments conducted with publicly available real datasets, which assess the validity and the reliability of the approach, in contrast to well-recognized methods; Section 6 summarizes the main outcomes extracted from the results. Section 7 outlines the fundamental conclusions derived from this work.

## 2. Vision System

**Figure 1:**
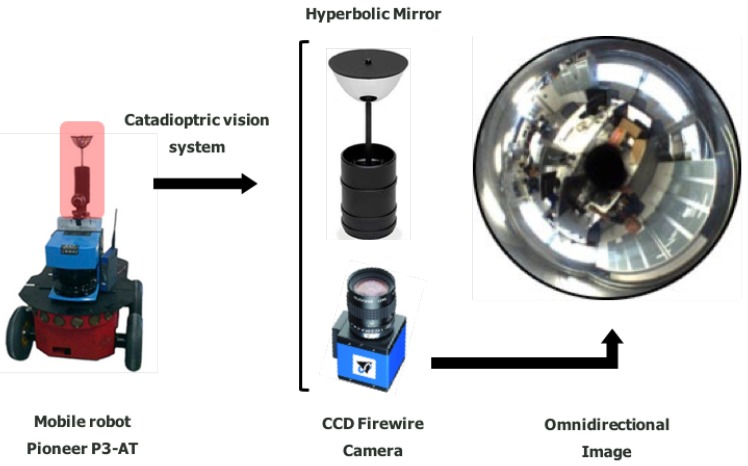
Real equipment constituted by a Pioneer P3-AT robot equipped with an internal odometer, a SICK-LMS200 laser range finder, and a catadioptric vision system, namely an omnidirectional vision system. This vision system is composed of a CCD (Charge-Coupled Device) FireWire DMK21BF04 camera, assembled with a hyperbolic Eizo h Wide 70 Mirror.

The equipment used in this work consists of a mobile robot, which is equipped with a laser range finder and a catadioptric vision system [31], as shown in Figure 1. The vision system is constituted by a hyperbolic mirror with a CCD (Charge-Coupled Device) camera jointly assembled. This represents a complete omnidirectional vision system, namely an omnidirectional camera.

**Figure 2:**
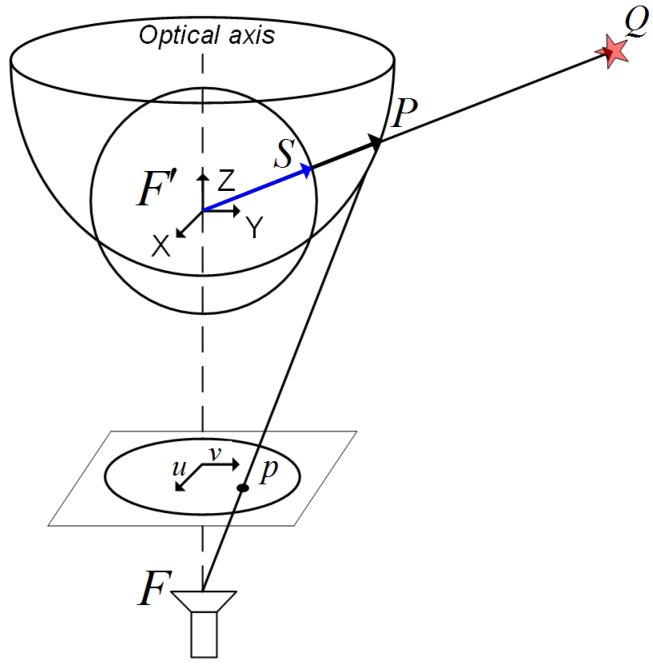
Omnidirectional camera 3D projection model from an XYZ-view.

According to [34], the projection model of our omnidirectional camera can be posed in terms of a central sphere projection system. Figure 2 reproduces the omnidirectional image generation in terms of such a projection, where a 3D scene point, $Q(x_{Q},y_{Q},z_{Q})\equiv Q$, is projected onto the mirror surface as *P*, onto the unitary sphere as *S*, and onto the camera plane as p(u,v)≡p. The focals of the hyperbolic mirror are *F* and $F^{\prime }$, *F* being coincident with the optical center of the CCD camera, whose optical axis lies in the *Z*-axis. Notice that the central sphere unifies the notation of the projection vectors for normalization purposes, according to the calibration of the omnidirectional camera [35], regardless of the characteristics of the mirror and its non-linearities. Thus the mapping of a 3D point onto the image plane can be analytically expressed as follows [34]:

(1) $$\rho \left[\begin{array}{c} p\\ a_{0}+a_{2}{\left||p|\right|}^{2}+\ldots +a_{n}{\left||p|\right|}^{n} \end{array}\right]=CQ$$

where *C*∈$R^{3\times 4}$ is the projection matrix, denoted as C=[R|T], with *R* a rotation matrix ∈$R^{3\times 3}$ and with $T=[t_{x},t_{y},t_{z}]$ a translation ∈$R^{3}$, between the camera and the global reference system. A Taylor expansion is introduced in order to model the effect of the mirror, whose coefficients ($a_{0},a_{2},\cdots ,a_{n}$) are experimentally estimated by a calibration toolbox [35]. Note that the monocular characteristic of this system leads us to include a scale factor, ρ = |T|.

## 3. Omnidirectional Visual Localization

The design of the localization model is constrained by the epipolar geometry [34] of two poses of the robot, from which two associated images are acquired. As in our former work [31], a conversion of the standard epipolar constraint is needed in this work in order to adapt it to the geometry of the omnidirectional system.

Solving the epipolar constraint implies estimating the essential matrix, $E_{3\times 3}$ [36], in order to extract the motion relation between two poses of the robot. To that aim, a set of matched points between the images acquired from these two poses, has to be introduced into the epipolar constraint:

(2) $$\begin{array}{c} {\tilde{x}}^{\prime T}E\tilde{x}=0 \end{array}$$

with $\tilde{x}$${}^{T}$ = ($x_{0},y_{0},z_{0}$) and $\tilde{x}$${}^{\prime T}$ = ($x_{1},y_{1},z_{1}$) being the normalized matched points expressed in the 3D global reference system, using the calibration of the vision system, which has been previously estimated [35].

The essential matrix *E* can be decomposed into a rotation *R* and a translation *T*, as denoted in Section 2. Assuming that the motion of our mobile robot is restricted to a 2D motion plane ∈ XY, *E* can be expressed by means of the skew symmetric ${\left[T\right]}_{x}$ and the mentioned rotation R:

(3) $$\begin{array}{cc} E={\left[T\right]}_{x}R & =\left[\begin{array}{ccc} 0 & 0 & \sin(\phi )\\ 0 & 0 & -\cos(\phi )\\ -\sin(\phi ) & \cos(\phi ) & 0 \end{array}\right]\left[\begin{array}{ccc} \cos(\beta ) & -\sin(\beta ) & 0\\ \sin(\beta ) & \cos(\beta ) & 0\\ 0 & 0 & 1 \end{array}\right]\\ & =\left[\begin{array}{ccc} 0 & 0 & \sin(\phi )\\ 0 & 0 & -\cos(\phi )\\ \sin(\beta -\phi ) & \cos(\beta -\phi ) & 0 \end{array}\right]=\left[\begin{array}{ccc} 0 & 0 & e_{1}\\ 0 & 0 & e_{2}\\ e_{3} & e_{4} & 0 \end{array}\right] \end{array}$$

where ${\vec{e}}_{i}=[e_{1},e_{2},e_{3},e_{4}$] stores the elements in *E*. Therefore, the motion relation can be recovered as a pair of rotation and translation angles (β,ϕ), between two poses of the robot, up to a scale factor ρ.

### 3.1. Angular Motion Recovery

More specifically, the retrieval of the rotation and translation angles, is expressed as the following linear system, which results from including Equation (3) into the epipolar constraint, expressed in Equation (2):

(4) $$D_{\mu x4}\cdot {\vec{e}}_{i}=\left[\begin{array}{cccc} x_{0}z_{1} & y_{0}z_{1} & z_{0}x_{1} & z_{0}y_{1} \end{array}\right]{\left[\begin{array}{cccc} e_{1} & e_{2} & e_{3} & e_{4} \end{array}\right]}^{T}=\vec{0}\;\forall i\in \left[1,\ldots ,N\right]$$

with μ being the total number of pairs of matching points, $\tilde{x}$${}^{T}=\left(x_{0},y_{0},z_{0}\right)$ and $\tilde{x}$${}^{\prime T}=\left(x_{1},y_{1},z_{1}\right)$. Consequently, for each μ-th pair of matching points, the μ-th row of Equation (4) constrains the angular motion by means of the elements in ${\vec{e}}_{i}$. It is worth noting that the system may be solved by using a minimum set of μ${}_{min}$ = 4 matching pairs. Finally, the application of Singular Value Decomposition (SVD) to Equation (4) allows us to compute the angular pair (β, ϕ). There is a quaternion of possible solutions that is eventually disambiguated by finding the positive ray’s intersection in front of the camera.

(5) $$\phi =a\tan\frac{-e_{1}}{e_{2}};\;\beta =a\tan\frac{e_{3}}{e_{4}}+a\tan\frac{-e_{1}}{e_{2}}.$$

**Figure 3:**
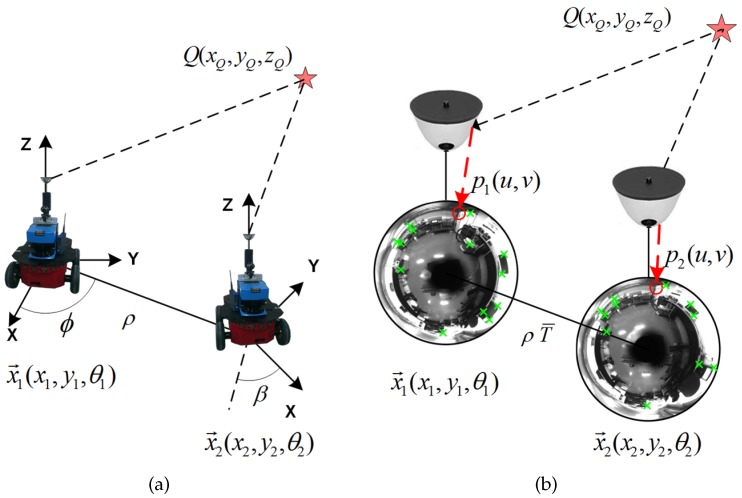
Omnidirectional visual localization between poses ${\vec{x}}_{1}$ and ${\vec{x}}_{2}$. (**a**) a 3D point $Q(x_{Q},y_{Q},z_{Q})$ is observed from the robot reference system; (**b**) additionally, the projections of *Q*, $p_{1}\left(u,v\right)$, and $p_{2}\left(u,v\right)$ are presented onto the camera reference system.

This angular motion, which finally determines the visual localization of the robot, is graphically depicted in Figure 3, where a univocal image-to-pose equivalence is presented. The same equivalence is expressed in the 3D robot reference system, in Figure 3a, and in the image reference system, in Figure 3b. A 3D point, $Q(x_{Q},y_{Q},z_{Q})$, is represented in the 3D robot reference system and its projections, $p_{1}\left(u,v\right)$ and $p_{2}\left(u,v\right)$, in the corresponding 2D image reference systems, captured from ${\vec{x}}_{1}\left(x_{1},y_{1},\theta_{1}\right)$ and ${\vec{x}}_{2}\left(x_{2},y_{2},\theta_{2}\right)$, which are ${\vec{x}}_{1}$ and ${\vec{x}}_{2}$, the 2D traversed poses, with orientation θ. The transformation between poses ${\vec{x}}_{1}$ and ${\vec{x}}_{2}$ is determined by the rotation R≡R(β), the translation T≡T(ϕ), and the scale factor ρ.

### 3.2. Scale Estimation

The lack of scale can be disambiguated by introducing certain visual patterns or specific objects with well-known 3D dimensions [11]. Since the 2D image projection for such objects or patterns can also be determined over different images, this leads to a triangulation problem [34] sustained by the epipolar constraint, where the 3D real dimensions and the 2D projections are known, and thus the variable to estimate is the scale factor, ρ. However, if such patterns or objects are not seen in the current frame for a long period of time, the estimation might be inaccurate. For this reason, we opted for using the scale estimate provided by the odometer, $\rho_{odo}$, which we input into the filter-based core system. Thus $\rho_{odo}$ is implicitly present into the prior measure of the filter, represented as the odometer’s control input, $u_{t}$, as detailed in Section 4.2. That permits obtaining updated estimates of the scale at every *t* and thus at the baseline between poses.

**Figure 4:**
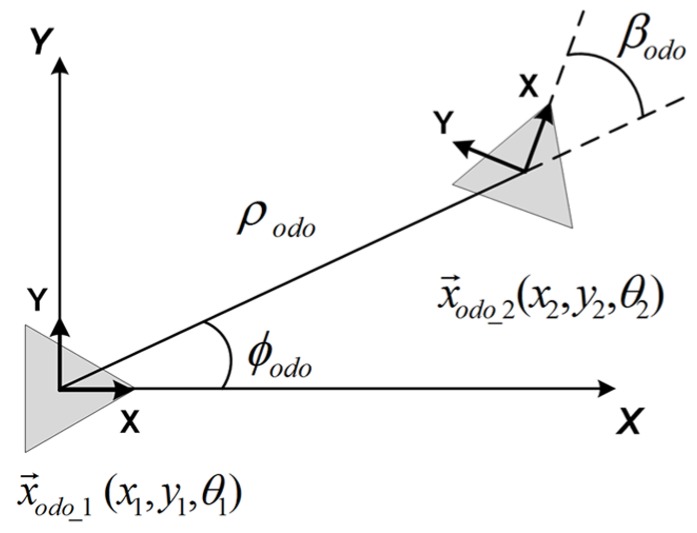
Odometer model.

The odometer provides readings ($\rho_{odo},\phi_{odo},\beta_{odo}$), which permit obtaining a relationship between two consecutive 2D poses traversed by the robot, expressed in the odometer’s notation as ${\vec{x}}_{odo_{1}}\left(x_{1},y_{1},\theta_{1}\right)$ and ${\vec{x}}_{odo_{2}}\left(x_{2},y_{2},\theta_{2}\right)$, being θ the orientation. As it may be observed in Figure 4, the relation between poses can be stated as follows:

(6) $$\left[\begin{array}{c} x_{2}\\ y_{2}\\ \theta_{2} \end{array}\right]=\left[\begin{array}{c} x_{1}\\ y_{1}\\ \theta_{1} \end{array}\right]+\left[\begin{array}{ccc} \cos(\phi_{odo})\; & \;0\; & \;0\;\\ \sin(\phi_{odo})\; & \;0\; & \;0\;\\ 0\; & \;1\; & \;1 \end{array}\right]\left[\begin{array}{c} \rho_{odo}\\ \beta_{odo}\\ \phi_{odo} \end{array}\right].$$

The error model for the odometer is parametrized by a probabilistic motion model [37], which adds zero-mean Gaussian noise, $N(0,\sigma^{2})$:

(7) $$\begin{array}{c} {\hat{\rho }}_{odo}=\rho_{odo}+\epsilon_{\rho }\;\;\to \;\;\epsilon_{\rho }\equiv N\left(0,\sigma_{\rho }^{2}\right) \end{array}$$

(8) $$\begin{array}{c} {\hat{\phi }}_{odo}=\phi_{odo}+\epsilon_{\phi }\;\;\to \;\;\epsilon_{\phi }\equiv N\left(0,\sigma_{\phi }^{2}\right) \end{array}$$

(9) $$\begin{array}{c} {\hat{\beta }}_{odo}=\beta_{odo}+\epsilon_{\beta }\;\;\to \;\;\epsilon_{\beta }\equiv N\left(0,\sigma_{\beta }^{2}\right). \end{array}$$

The standard deviations required to complete the parametrization are computed by using the empiric parameters provided by the manufacturer ($\alpha_{1},\alpha_{2},\alpha_{3},\alpha_{4}$), as follows:

(10) $$\begin{array}{cc} \sigma_{\rho }= & \alpha_{3}\rho_{odo}+\alpha_{4}\left(\right|\phi_{odo}\left|+\right|\beta_{odo}\left|\right) \end{array}$$

(11) $$\begin{array}{cc} \sigma_{\phi }= & \alpha_{1}\left|\phi_{odo}\right|+\alpha_{2}\rho_{odo} \end{array}$$

(12) $$\begin{array}{cc} \sigma_{\beta }= & \alpha_{1}\left|\beta_{odo}\right|+\alpha_{2}\rho_{odo}. \end{array}$$

### 3.3. Notation Definitions

In this subsection, the notation of the localization method is presented. We define a state vector, s(t), that comprises the different variables implied in the estimation. This state vector stores a set of consecutive poses of the robot which are estimated by the localization method. These poses are the result of discretizing the trajectory traversed by the robot. Assuming that an omnidirectional image is captured from a certain 2D pose ${\vec{x}}_{i}$(x,y,θ), θ being the robot’s orientation, such an image can be denoted as a view, encoded as a set of SURF feature points [23] that are extracted from it. The pose of each view is included in the state vector as ${\vec{x}}_{n}=(x_{n},y_{n},\theta_{n}{)}^{T}$, ∀n∈[1,⋯,N]. The current pose of the robot at time *t* is expressed as ${\vec{x}}_{t}=(x_{t},y_{t},\theta_{t}{)}^{T}$. Thus the definition of the state vector includes ${\vec{x}}_{t}$ and ${\vec{x}}_{n}$, with the following 2D structure:

(13) $$s\left(t\right)={\left[\begin{array}{cccccc} {\vec{x}}_{t} & {\vec{x}}_{1} & \cdots & {\vec{x}}_{n} & \cdots & {\vec{x}}_{N} \end{array}\right]}^{T}.$$

Therefore, the state vector comprises a trajectory with a total number of *N* views. These variables represent a dual encoding model of the environment. They are expressed in 2D, due to the fact that we work with a robot that is assumed to move in a 2D plane. However, given a specific calibration [35] and the estimation of the scale factor, every 2D point detected inside the views can be back-projected to the 3D global reference system by means of Equation (1). Therefore, re-estimating a view, implies that the entire 3D information of the map is re-estimated at once. This aspect makes the approach more efficient than traditional 3D landmark models [11,38], which need the 3D re-estimation of every single landmark at every *t*.

Considering this framework within the field of mobile robotics, it is also worth defining a formal observation model, which permits estimating the localization. The procedure has been detailed in the previous subsection. However, it is expressed here in accordance with the state vector’s variables, s(t). The motion relation between two poses of the robot can be retrieved in an angular format, as in Equation (5). Transferring the angular localization relation (β,ϕ) into the robot’s reference nomenclature, the following observation measurement can be established:

(14) $$\begin{array}{c} z_{t,n}=\left[\begin{array}{c} \phi \\ \beta \end{array}\right]=\left[\begin{array}{c} \arctan\left(\frac{y_{n}-y_{t}}{x_{n}-x_{t}}\right)-\theta_{t}\\ \theta_{n}-\theta_{t} \end{array}\right] \end{array}$$

where the notation corresponds to the one expressed in Equation (13), and thus $z_{t,n}$ represents the angular motion between the current pose of the robot, ${\vec{x}}_{t}$, and a view in the state vector, ${\vec{x}}_{n}$.

## 4. Visual Information Fusion

Once the localization model has been described, this section introduces the implementation of the visual information fusion into the system and the rest of the details associated with the main contribution.

**Figure 5:**
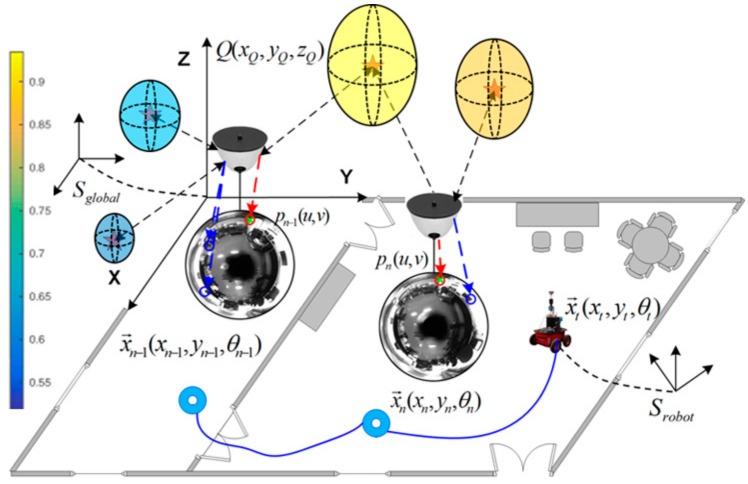
Robot navigation example in an office-like scenario along three poses: ${\vec{x}}_{n-1}$, ${\vec{x}}_{n}$, and ${\vec{x}}_{t}$. The 3D probability distribution of feature points’ existence permits associating visual feature points with a specific probability, indicated with colored spheres, whose probability values are encoded according to the left-side colorbar. Projections of a 3D point $Q(x_{Q},y_{Q},z_{Q})$, $p_{n-1}\left(u,v\right)$ and $p_{n}\left(u,v\right)$, are also indicated. The 3D global reference system is denoted as $S_{global}$, and the 3D robot reference system as $S_{robot}$.

The main goal is to obtain a model that accounts for the visual changes in the environment and encodes the probability of existence of visual feature points in the 3D global reference system. Figure 5 illustrates an example of this idea, where the 3D environment is modeled with a specific probability of feature existence. This approach will be extended in order to predict spatial areas from which visual information is more likely to be detected. Such areas can be also projected onto the next image frame so as to map pixel areas where matching is more likely to appear, rather than in other parts of the image, in terms of probability.

A first sketch might consist in recording statistics of feature points that are tracked along the navigation of the robot through the environment. This would lead us to infer a probability distribution for the existence of 3D points along the trajectory of the vehicle, in terms of percentage of occurrence. Nonetheless, a more precise formulation can be introduced as follows, by using Bayesian inference.

**Figure 6:**
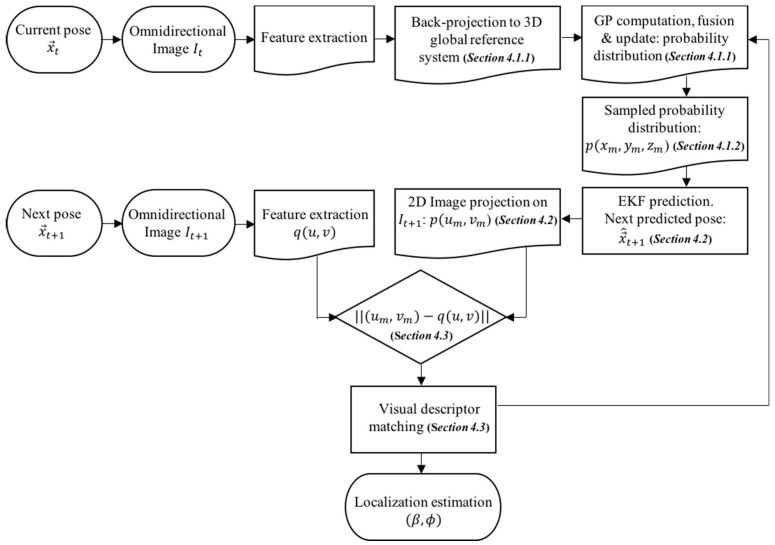
Block diagram of the presented approach.

A general overview of the entire process can be observed in Figure 6. The main contributions, which try to obtain a probability-oriented feature matching, are present in the following blocks: the 3D back-projection, the GP computation to produce the 3D probability distribution, the probability sampling, and the 2D image projection over the next predicted pose.

### 4.1. 3D Probability Distribution of Feature Existence: GP Computation and 3D Probability Sampling

#### 4.1.1. GP Computation

In this work, we use the same Bayesian regression technique applied in [31], formulated as a Gaussian Process (GP) [32]. However, in this approach, we pursue the probability distribution of feature points’ existence in the 3D global reference system rather than in the 2D image frame. GP is able to produce reliable regression results without the need of common associations between inputs and outputs, in comparison to traditional inference techniques [33]. Its general notation is the following:

(15) $$f\left(x\right)∼\mathrm{GP}[m\left(x\right),k\left(x,x^{\prime }\right)]$$

where the GP function is expressed as f(x), with mean m(x) and covariance $k(x,x^{\prime })$. The training and test input points, *x* and $x^{\prime }$, respectively, represent 3D points at which the value of the function is tested in terms of probability of existence.

Since we intend to obtain a probability distribution in 3D, the nomenclature for the output function has to be adapted to the formulation of our approach, so f(x)≡f[X(x,y,z)], X(x,y,z) being a general 3D point in the global reference system, such that $X\left(x,y,z\right)\equiv X_{global}$. Thus f(·) evaluates the probability of existence of a feature point over a 3D point in the space. Then f(·)∈ [0,1].

The input for the GP is represented by the feature matching between two images associated with two poses traversed by the robot, up to time *t*. As mentioned above, the Bayesian inference requires data in the 3D global reference system, so the 2D feature matching has to be back-projected to the 3D global reference system. As initially presented in Equation (1), this transformation can be achieved thanks to the scale factor estimation, and the specific camera calibration [35], which establishes the conversion between the 2D image frame and the 3D global reference system.

There is a final step before obtaining the exact 3D global reference system representation. The previous back-projection is expressed in the current 3D robot reference system, therefore we apply the following expression in order to formally convert to the proper 3D global reference system.

(16) $$X_{global}=\rho T+RX_{robot}$$

where a 3D point expressed in the current 3D robot reference system, as $X_{robot}$, is transformed into the 3D global reference system, expressed as $X_{global}$, by means of the rotation, *R*, translation *T*, and scale factor ρ, presented in Section 3, according to the localization measures.

#### 4.1.2. 3D Probability Sampling

**Figure 7:**
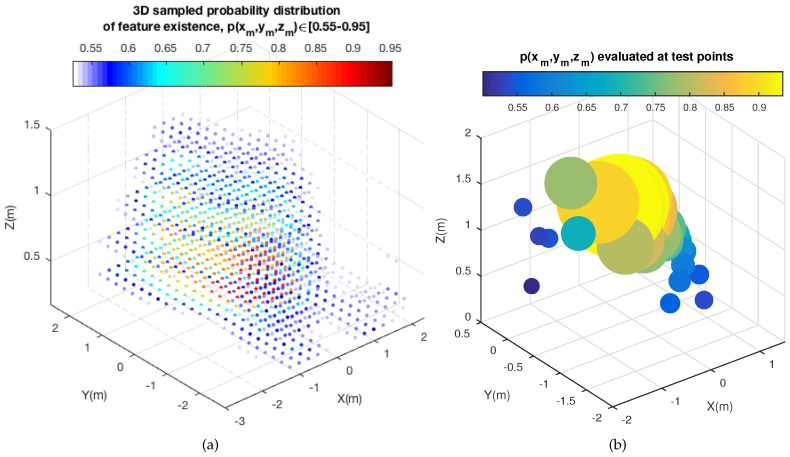
3D sampled probability distribution of feature existence. (**a**) Complete 3D sampled probability distribution, $p(x_{m},y_{m},z_{m})$; (**b**) $p(x_{m},y_{m},z_{m})$ evaluated at the last feature points observed (test points).

At this point, we have obtained a 3D probability distribution of feature existence up to time *t*, denoted as f[X(x,y,z)]. The next step seeks the reduction of computational resources. To that purpose, a 3D sampling over f[X(x,y,z)] has to be devised in order not to compromise the computational resources of the system. In consequence, a normalization of the 3D probability distribution is carried out. Then, the sampling discretization corresponds to a 3D square grid, as follows:

(17) $$\begin{array}{c} P_{acc}={\;\int \int \int \;}_{V}f\left[X\left(x,y,z\right)\right]\;dx\;dy\;dz \end{array}$$

(18) $$\begin{array}{c} f_{norm}\left[X\left(x,y,z\right)\right]=\frac{f[X(x,y,z)]}{P_{acc}} \end{array}$$

(19) $$\begin{array}{c} p_{norm}=\sum_{x_{m}}^{M}\sum_{y_{m}}^{M}\sum_{z_{m}}^{M}f_{norm}\left(x_{m},y_{m},z_{m}\right)=1\;m\in \left[1,M\right] \end{array}$$

(20) $$\begin{array}{c} p\left(x_{m},y_{m},z_{m}\right)\equiv f_{norm}\left(x_{m},y_{m},z_{m}\right) \end{array}$$

where $P_{acc}$ is the total accumulated probability, which is computed for normalization purposes, so as to obtain $f_{norm}\left(\cdot \right)$. Then the definition of the 3D square grid, with $M^{3}$-elements, allows us to obtain a sampled normalized probability distribution, $p(x_{m},y_{m},z_{m})$. Additionally, Figure 7 presents a real example of a 3D sampled probability distribution of feature points’ existence. Figure 7a shows the complete sampled distribution, $p(x_{m},y_{m},z_{m})$, whereas Figure 7b shows the evaluation of such a distribution at the last feature points observed, as test points, after being back-projected from the 2D image frame to 3D. Notice that, for better illustration, high probability values are represented with a higher radius. These data will be fused into the distribution as the next input data that the GP uses to update the current distribution. The axes represent the 3D global position within the sampling grid $\left(x_{m},y_{m},z_{m}\right)$ and the 3D probability of feature existence at such positions. $p(x_{m},y_{m},z_{m})$ is expressed by a gradient of color.

### 4.2. Motion Prediction and 2D Image Projection

Since the main goal is to predict relevant areas in the 2D image frame, where feature matching is more likely to appear, the resulting 3D probability distribution obtained by GP up to time *t*, $p(x_{m},y_{m},z_{m})$ after sampling, has to be projected onto the next 2D image frame in time t+1, associated with the next pose of the robot. Therefore, the configuration of a prior prediction stage is essential. In this sense, we take the most of an EKF (Extended Kalman Filter)-based filter formulation, similarly to [31,39].

After customizing this configuration properly, we are able to predict the next pose ${\hat{\vec{x}}}_{t+1}$. As detailed in Section 3.2, the scale factor is disambiguated as the estimate provided by the odometer, $\rho_{odo}$. It is worth noting that this value is present in the odometer’s control input, $u_{t}\equiv f\left(\rho_{odo},\phi_{odo},\beta_{odo}\right)$, which represents the prior input for the EKF-based system. This can be observed in the notation of the EKF-based prediction stage, listed in Table 1.

**Figure 8:**
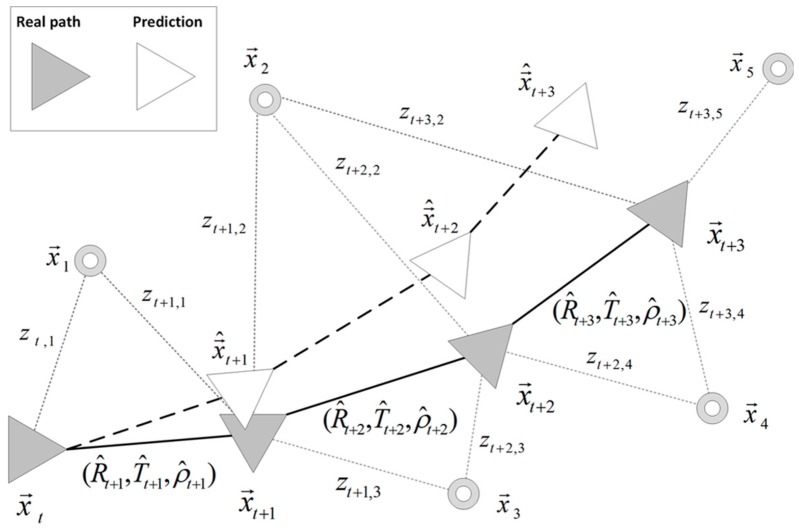
Graph diagram of a robot trajectory. Real path poses, ${\vec{x}}_{t}$, and predicted poses, ${\hat{\vec{x}}}_{t}$, at each *t* are indicated, following the notation described in Equations (13) and (14). Observation measurements, $z_{t,n}$, and views in the environment, ${\vec{x}}_{n}$, are also depicted.

Thereby we are able to decompose [34] the predicted motion from pose ${\vec{x}}_{t}$ to ${\hat{\vec{x}}}_{t+1}$, in a rotation $\hat{R}$ and a translation $\hat{T}$.

(21) $$\hat{R}∼N\left(\hat{\beta },\sigma_{\beta }\right);\;\hat{T}∼N\left(\hat{\phi },\sigma_{\phi }\right)$$

where $\sigma_{\beta }$ and $\sigma_{\phi }$ are the standard deviations that characterize the proposed localization method described in Section 3. Figure 8 synthesizes the motion prediction process, where $z_{t,n}$ represents the observation measurement of a certain view in the environment, ${\vec{x}}_{n}$, computed from the current pose, ${\vec{x}}_{t}$, as described in Equations (13) and (14).

**Figure 9:**
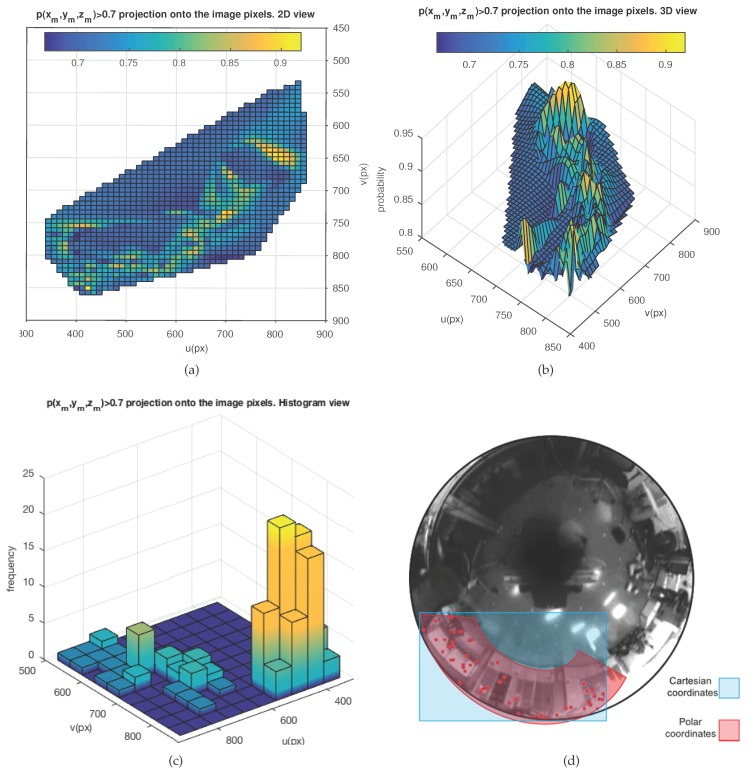
Projection of the 3D sampled probability distribution of feature existence, $p(x_{m},y_{m},z_{m})\in$ [0.7–1], onto the image pixel axes, in *t*. (**a**) 2D representation, $p(u_{m},v_{m})$. (**b**) 3D representation with *Z*-axis expressing probability, $p(x_{m},y_{m},z_{m})$. (**c**) 2D histogram representation. (**d**) Euclidean versus polar coordinates.

Finally, the 3D probability distribution $p(x_{m},y_{m},z_{m})$ is projected onto the pixels of the 2D image frame associated with the next pose of the robot, denoted as $p(u_{m},v_{m})$, by applying Equation (1). Furthermore, a specific probability range with custom values can be defined, [$p_{min}$–$p_{max}$], in order to only select points from the distribution with probabilities within that range. The immediate outcome is the generation of probability areas in the image frame, where feature matching is more likely to appear. Figure 9 presents a real example after applying the overall method to the image acquired from the current robot pose. The visualized probability range is p∈ [0.7–1]. Figure 9a represents the projection of $p(x_{m},y_{m},z_{m})$ onto the image plane, as $p(u_{m},v_{m})$ in 2D, and Figure 9b in 3D. Figure 9c presents the same 2D projection after transforming the axes in order to generate a histogram representation. This last representation is useful for data processing tasks. Finally, Figure 9d reveals that polar space encoding might produce a better modeling of the distribution rather than Cartesian coordinates. This may be implicitly induced from the elliptical constitution of the epipolar curves in an omnidirectional vision system.

### 4.3. Probability-Oriented Feature Matching

The final stage is intended to perform feature matching. Using the method presented in the previous subsection, probability areas can be detected on the image. Considering this, a straightforward design would entail using a feature detector only on the desired areas, and thus filtering by high probabilities. This would avoid processing the entire image. Nevertheless, it would lead to errors, under certain circumstances, especially when the robot discovers new scenes in the environment. If we assume that there may be substantial changes in the visual appearance as the robot goes through new areas, then it will be necessary to let these new areas be processed in order to detect new features. This is the main reason why we keep detecting features all over the images, so that we allow the GP to update its output when new visual content is discovered. Otherwise the visual content of these new scenes would never be fused into the probability of feature existence, computed by GP.

Taking these last considerations into account, we measure the proximity between the pixels, $\left(u_{m},v_{m}\right)$, associated with the sampled projected probability distribution on the 2D image frame, $p(u_{m},v_{m})$, and all the feature points detected in the next image, q(u,v). Such proximity is computed by means of the Mahalanobis distance [40], $\left|\right|\left(u_{m},v_{m}\right)-q\left(u,v\right)\left|\right|$. Those feature points in q(u,v) are accepted as matching candidates when their pixel distance to $\left(u_{m},v_{m}\right)$ meets the confidence threshold established by the chi distribution, χ(dof), evaluated at the degrees of freedom that represent the dimensionality of the involved variables. Since the image frame is defined at a pixel level, the degrees of freedom are dof = dim(u,v) = 2.

(22) $$\left|\right|\left(u_{m},v_{m}\right)-q\left(u,v\right)\left|\right|\leq \chi \left[dim(u,v)\right].$$

**Figure 10:**
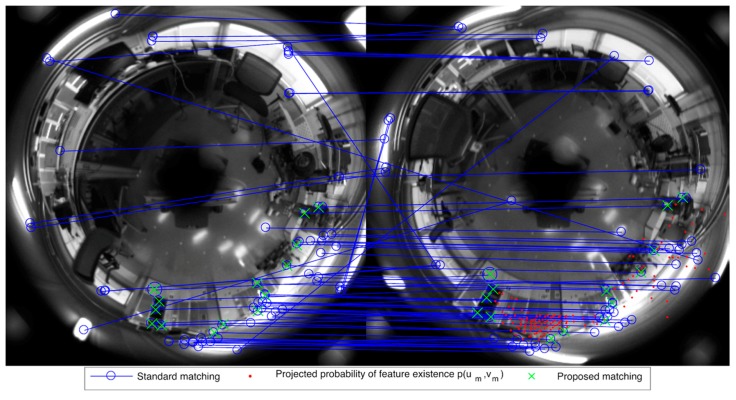
Matching results between images acquired from poses at *t* and t+1. Standard matching results are indicated with blue circles, and those obtained with the proposed approach are indicated with green crosses. The pixels associated with the projected probability of feature existence $p(u_{m},v_{m})$ are indicated with red dots.

The feature points in q(u,v) that meet Equation (22), namely matching candidates, are then matched through a standard matching process by visual descriptor comparison. In the end, these matching points are the final data which will be used in the localization system in order to obtain an estimation of the current pose of the robot, as previously introduced in Section 3. Finally, the same real example presented in Figure 9 is further detailed in Figure 10, over the corresponding real omnidirectional images, between poses at *t* and t+1. Here, the proposed approach is compared with a standard matching block [23]. It can be observed how the standard matching (blue circles) produces a significant amount of false positives. Our proposal produces a set of valid matching points (green crosses) under the constraint of the probability area represented by its projection on the image (red dots).

Regardless of the smaller set of matches obtained, we can rely on the consistency and robustness of these points, since they are highly probable according to the current navigation of the robot. Even under a hypothetical situation where no match is obtained, we can rely on the filter-based estimation until new matches are detected in a subsequent frame. Moreover, as already mentioned, there is no restriction for other new feature points to be matched. A modulation of the selected probability range p∈ [$p_{min}$–$p_{max}$] is achieved through an adaptive scheme, which is referred to as the current uncertainty of the entire probability distribution $p(x_{m},y_{m},z_{m})$. Similarly to [31], we assess the drifts on uncertainty by evaluating the information gain, using the information-based Kullback–Leibler divergence (KL) [41], with the aid of the standard entropy metric [42]. In this manner, when there are considerable changes in the visual appearance of the scene, the probability distribution produced by GP will change accordingly. The KL measure will encode such change, which will lead the system to modulate the desired range p∈ [$p_{min}$–$p_{max}$], in order to adapt the final matching to the current uncertainty conditions of the system. Nonetheless, and in contrast to [31], this approach encodes fluctuations in the probability expressed in a 3D global reference system, rather than in the particular 2D image frame of each pose. Furthermore, its application is also different, since we use it to modulate the custom probability ranges, rather than using it as a weighting coefficient for the matching.

## 5. Results

This section presents a set of experiments conducted with publicly available datasets [43,44]. They assess and compare the performance of the matching and the visual localization, in comparison with well-acknowledged methods [6,23,45]. A public benchmark toolkit [44,46] has also been used to produce such comparisons. Table 2 comprises the characteristics of the datasets.

The computation specifications of the real equipment presented in Figure 1 are: CPU 2 × 1.7 GHz; RAM 2 Gb. The acquisition of omnidirectional images (1280 × 980 px) demands the system to compute estimates at 1.5 Hz. The SICK-LMS200 laser data are utilized to obtain a ground truth estimation for comparison purposes. Finally, the robot is run by the operating platform ROS [47].

### 5.1. Matching Results

The first set of experiments was conducted with the Innovatrajectory dataset, in order to evaluate the capability of the approach to produce robust probability-oriented matching results. Each subset within these results’ series was configured with a 300-times execution setup, so as to obtain consistent average results. The first set of results evaluates the matching performance between poses of the robot, from which omnidirectional images were captured.

This performance is evaluated through the number of feature matches, the accuracy, and the computation time.

#### 5.1.1. Number of Feature Matches

**Figure 11:**
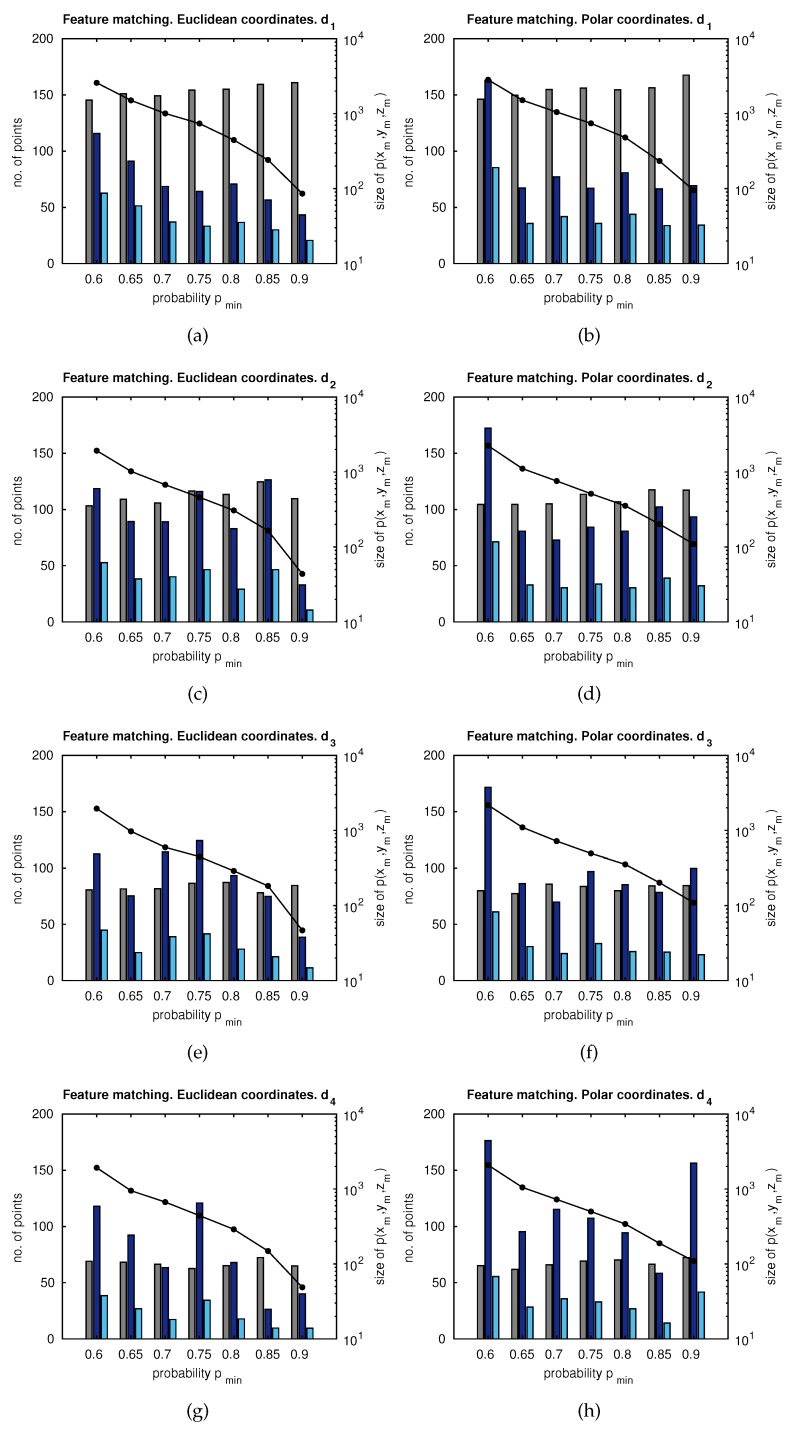
Left axes: number of matches versus $p_{min}$. Right axes: size of the probability distribution (log) versus $p_{min}$. −•− size[$p(x_{m},y_{m},z_{m})$]. Euclidean coordinates and distance between capture points: (**a**) $d_{1}$; (**c**) $d_{2}$; (**e**) $d_{3}$; (**g**) $d_{4}$. Polar coordinates and distance between capture points: (**b**) $d_{1}$; (**d**) $d_{2}$; (**f**) $d_{3}$; (**h**) $d_{4}$. Legend: ■ Standard matching; ■ proposed matching candidates; ■ proposed final matching.

Figure 11 presents matching results with different configurations. The *X*-axis represents the minimum range for the probability of feature existence, $p_{min}$. The distance between consecutive poses has been considered a variable parameter ($d_{1}$ to $d_{4}$), being $d_{i}=0.25i$ meters, and the influence of this distance was tested. The left-side *Y*-axis represents the number of features obtained by a standard matching technique [23] (grey), the proposed matching candidates (dark blue), and the final number of matches of the proposed approach (light blue). The right-side *Y*-axis (log) represents the size of $p(x_{m},y_{m},z_{m})\;$<$\;p_{min}$. Additionally, the influence of using either Euclidean coordinates (left column) or polar coordinates (right column) was assessed.

Figure 11 evidences that higher distances between images produce a lower number of matching points, considering both the standard matching and the proposed approach. Despite this fact, our proposal provides more matching candidates than the standard approach. Moreover, the drop in the final number of matching points is less accentuated in the proposal, thus ensuring a minimum of matching points, even when images are captured from distant poses. Polar coordinates produce more matches only when $p_{min}$ is high. In other words, when the size of $p(x_{m},y_{m},z_{m})$ is low, the probability areas on the image, where matches may be found, are reduce. In any other case, Euclidean coordinates are more suitable due to their good balance between computational cost and the amount of matching data. According to these results, only Euclidean coordinates and extreme distances, $d_{1}$ and $d_{4}$, are considered.

#### 5.1.2. Accuracy

**Figure 12:**
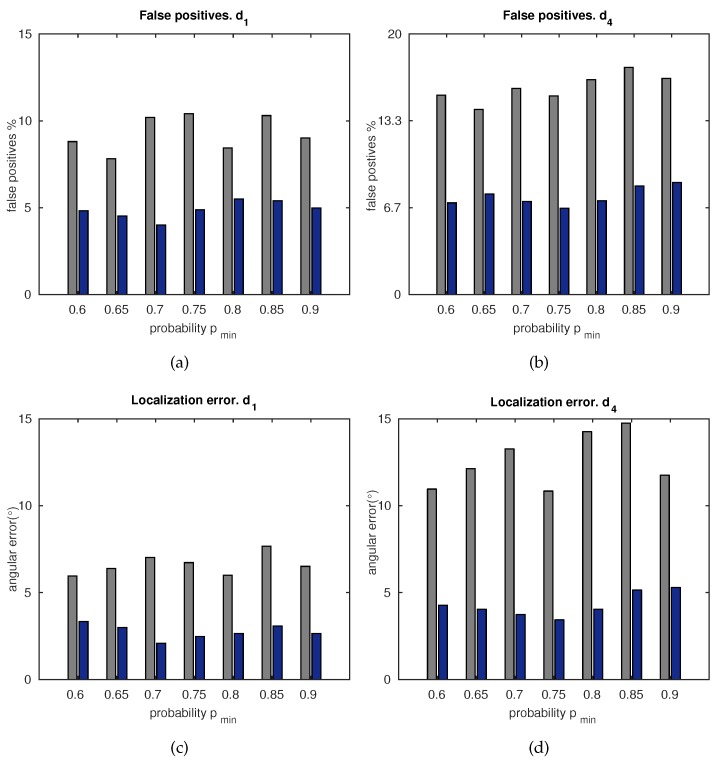
Top row: percentage of false positives. (**a**) Distance $d_{1}$; (**b**) distance $d_{4}$. Bottom row: localization error (in β and ϕ) versus $p_{min}$. (**c**) Distance $d_{1}$; (**d**) distance $d_{4}$. Legend: ■ standard matching; ■ proposed matching.

The accuracy of the approach is compared with a standard matching technique [23] in Figure 12. Figure 12a,b show the percentage of false positive matches obtained with the standard matching (grey) and the proposed matching (dark blue), respectively. In the same manner, Figure 12c,d compare the resulting localization error (mean of β and ϕ), according to Equation (5) for the standard matching (grey) and the proposed matching (dark blue). All these results show correlated errors due to the fact that the same percentage of false positives is still present in the localization computation.

Besides this, the error decreases with $p_{min}$, up to an intermediate value, after which it rises slightly. This is due to the fact that high values of $p_{min}$ may restrict the probability of feature existence on the image, to a set of few areas which may be closely arranged. Hence, this may lead the system to focus only on narrow areas of the image, dismissing newer visual information. Although this effect was considered in Section 4, and in the modulation of p∈ [$p_{min}$–$p_{max}$] by the KL divergence, there is still a subtle influence that can be observed in Figure 12. As a result, it is worth configuring the system with intermediate values such as p∈ [0.65–0.75], and then modulating its limits within that range, by means of the evaluation of the current uncertainty through the KL divergence.

#### 5.1.3. Computation Time

**Figure 13:**
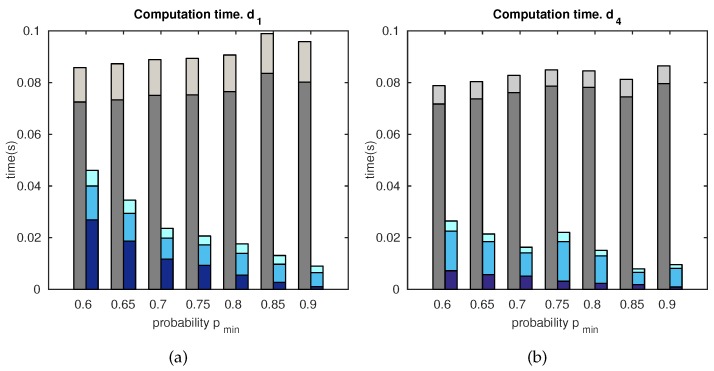
Computation time versus $p_{min}$. (**a**) Distance $d_{1}$; (**b**) distance $d_{4}$. Legend: ■ standard matching: matching computation; ■ standard matching: localization computation; ■ proposed matching: candidates’ computation; ■ proposed matching: matching computation; ■ proposed matching: localization computation.

Figure 13 compares the computation time for the standard matching and the proposed matching, versus $p_{min}$, and distances $d_{1}$, $d_{4}$. The total computation time has been divided into different parts, as indicated in the legend, in order to determine the different contributions:

(a) feature matching;
(b) matching candidates;
(c) final localization estimation.

These results are closely related to those presented in Figure 11, since the standard matching and the proposed matching, spend less time when the number of matches is lower. This permits selecting a suitable tradeoff solution between computation and accuracy, as $p_{min}$ = 0.7. In addition to this, the proposed approach is shown to produce more efficient results than the standard matching technique.

### 5.2. Localization Results

**Figure 14:**
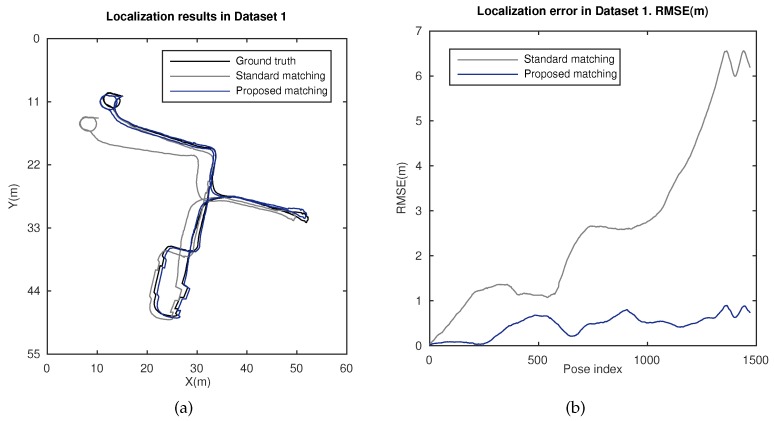
Localization results in Dataset 1, Innovatrajectory. (**a**) Localization estimation obtained with ground truth (black), standard matching (grey), and the proposed matching (blue); (**b**) RMSE (m) for the localization estimation with standard matching (grey) and the proposed matching (blue).

This section deals with the localization estimation, produced by this approach. Figure 14 presents localization results for the Innovatrajectory dataset. Figure 14a shows a bird’s eye view of the estimated poses (plane XY, meters). Estimations obtained with the standard matching [22,23] and the proposed matching are compared. Figure 14b carries out the same comparison in terms of the root mean square error (RMSE), for both approaches. It can be observed that our proposal outperforms the localization method with standard matching and is capable of ensuring a bounded error at every *t*, in contrast to the standard method error, which increases substantially with *t*. These results validate the design of this approach and its performance, according to the previous subsection.

**Figure 15:**
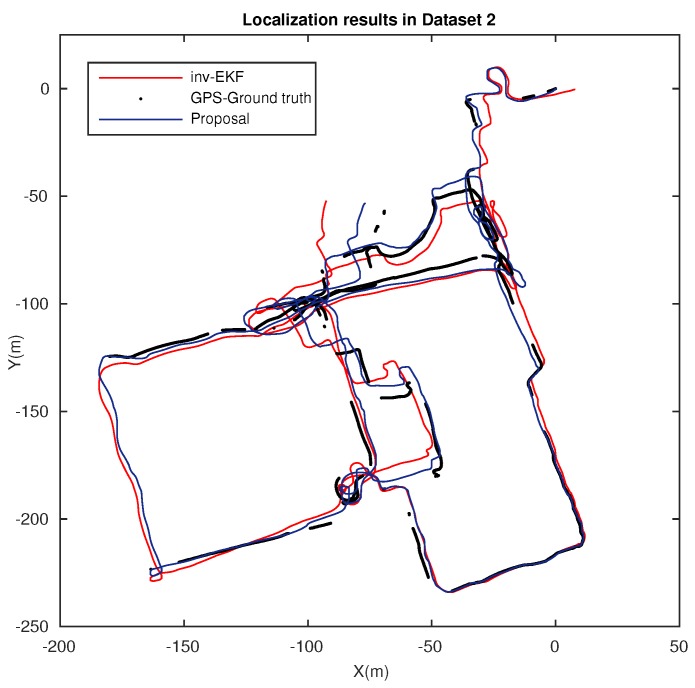
Localization results in Dataset 2, *Bovisa 10-04-2008*. Localization estimation obtained with ground truth (GPS) (black), inverse EKF (red), and the proposed matching (blue).

In addition to the previous localization results, we compared our method with a widely recognized approach, the inverse EKF with depth parametrization [38]. To that end, we used the benchmark toolkit, publicly available in [44], and which also provides Dataset 2, *Bovisa 2008-10-04*. The results generated by the inverse EKF technique can be further consulted in [6,45]. Figure 15 presents localization results in a very challenging outdoor scenario, where dynamic conditions are highly relevant and challenging. Localization estimation results for the inverse EKF (red), the proposal (blue), and the zones where ground data (GPS) are available (black) are depicted. At first inspection, our approach demonstrates an improved reliability.

**Figure 16:**
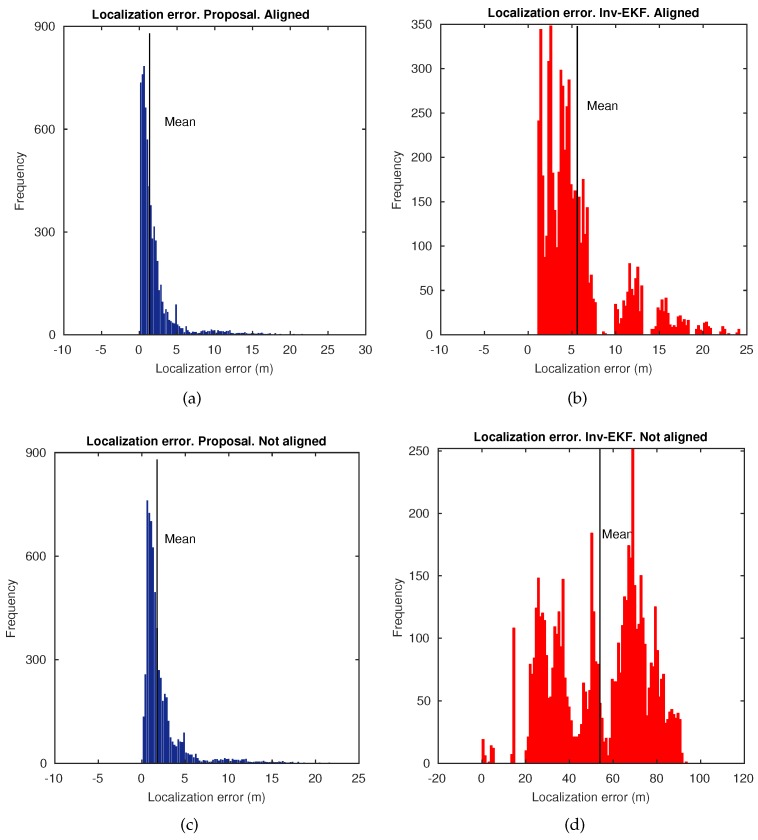
Localization error histograms in Dataset 2, *Bovisa 10-04-2008*. (**a**) Proposed approach with alignment enabled. (**b**) Inverse EKF approach with alignment enabled. (**c**) Proposed approach with alignment disabled. (**d**) Inverse EKF approach with alignment disabled.

Moreover, further accuracy results are provided in Figure 16, where histograms of the error at each estimated pose in *t*, are presented. Two different setups have been considered to obtain these histograms. The inverse EKF does not disambiguate the lack of scale [6,45]. That is the reason why the final estimate only confers reliability on its topological form with respect to the ground truth, but not on its metric form. According to this, the benchmark toolkit provides a Maximum Likelihood Estimator (MLE) that can be applied in order to align the final estimated trajectory, and thus overcoming this issue. Hence, we can enable/disable this alignment method. Therefore, Figure 16a,c, represent the error histograms for the proposed approach, with alignment enabled and disabled, respectively. In the same manner, Figure 16b,d, represent the error histograms for the inverse EKF. It can be noted that the proposed approach improves the accuracy results in contrast to the inverse EKF, regardless of the operation of the alignment method, with average localization errors under 2 m.

## 6. Discussion

This section analyzes the main aspects regarding the implications extracted from the results. Initially, Figure 11 revealed the capability of the approach to provide probability-oriented matching points, which meet a specific probability distribution of feature existence, according to the Bayesian inference provided by GP. Despite the fact that increasing the distance between capture points implies a substantial decrease on the number of matches found, this proposal proves to keep a stable amount of valid matches, even at long distances, contrarily to a standard matching.

A similar deduction can be made by inspecting Figure 12. In this figure, false positives and localization errors are assessed, and a robust matching procedure is confirmed. Considering that the matching data are then processed into the localization system, it is evident that these results are closely correlated. This approach confirms a good and stable accuracy under the worst situation expected in a matching process, that is, under the presence of false positives. It is worth noting the effect of varying $p_{min}$. High values of $p_{min}$ may lead the system to narrow on a reduced set of probability areas over the image. This fact may also imply that the visual information contained in new visual spaces discovered by the robot, is dismissed. However, we took this issue into account in order to modulate $p_{min}$. To that purpose, the localization system is set to work autonomously and computes the information divergence, KL, as a measure of the drifts of the uncertainty of the system. Despite this fact, a subtle influence of this effect is still present, and it can be noticed in the figures. Therefore, an optimal configuration can be selected with values within $p_{min}\in$ [0.65–0.75].

To complete the analysis, the computational costs required by this approach were evaluated. Figure 13 demonstrates that the proposal can be adequately tuned in order to confer valid and robust estimates, which permit working in real time. A relaxed tradeoff can be easily established between accuracy and computation resources. This approach proves to be a more efficient solution than a standard matching technique, at every studied aspect.

Finally, the outcomes of this work have been evaluated in terms of the localization performance. Figure 14 presents suitable results in a large indoor environment. A reliable and robust operation is ensured with stable error, in contrast to the performance offered by a standard matching. Furthermore, the results of a well-acknowledged method are presented in Figure 15 for comparison. Once again, the validity and robustness of our approach in terms of the accuracy of the final estimation, regardless of the challenging conditions in such environment, are reinforced.

Summarizing, the following achievements can be highlighted:

- Adaptive probability-oriented feature matching.
- Stable amount and accurate matches provided, in contrast to standard techniques.
- Efficient approach to work in real time.
- Robust final localization estimate in large and challenging scenarios.

## 7. Conclusions

This work has presented an information fusion approach for robust probability-oriented feature matching. It uses an omnidirectional vision system for visual localization purposes, and it is an improved extension of [31]. The approach is sustained by visual data fusion through Bayesian inference. The real system is constituted by a mobile robot, equipped with a monocular omnidirectional vision system, which is adequately adapted to work under the constraint of the epipolar geometry between images.

The main goal was to produce a robust approach to obtain relevant and reliable matching points for further localization tasks. To that end, several contributions were designed and implemented. Firstly, the 3D visual information associated to feature points is inferred by a Bayesian technique, represented by GP. Its output, at every *t*, provides a 3D probability distribution of feature existence in the global reference system. This probability is successively fused and updated while the robot navigates. Secondly, a normalization and sampling is produced in order to alleviate the computation requirements. After that, by taking most of the EKF prediction stage, the sampled probability can be projected in the next 2D image frame, at t+1. This is the last step that allows us to map relevant areas in the next image, from which matches with high probability of appearance are expected.

The principal output of the implemented contributions is a dynamic model that adapts the matching according to the visual changes on the scene, by introducing formal probability definitions. The approach has demonstrated to adequately balance the matching between highly relevant areas, in terms of current probability, and new visual spaces discovered by the robot. This has been achieved by modulating the probability areas on the image, by a KL metric over the uncertainty of the system.

The benefits of the contributions presented in this approach have been reinforced by the results obtained with real data, computed with publicly available datasets. The suitability and robustness of the matching proposal have been demonstrated with performance tests, in terms of accuracy and efficiency, in comparison with standard matching techniques. Furthermore, its performance has been further evaluated under a visual localization context, in both large indoor and outdoor scenarios. It has also been shown to outperform a well-acknowledged localization method (the inverse EKF). These results have confirmed the validity and consistency of the proposed approach.

## Figures and Tables

**Table 1:** EKF-based Filter: Prediction stage.

Filter-Based SLAM Stages		
Stage	Expression	Terms
**Prediction**	${\hat{\vec{x}}}_{t+1|t}=f_{t}({\hat{\vec{x}}}_{t|t},u_{t})$	$f_{t}$: relates the odometer’s control input $u_{t}$ and the current state
	${\hat{z}}_{t+1|t}=h_{t}({\hat{\vec{x}}}_{t+1|t},{\vec{x}}_{i})$	$u_{t}$: odometer’s control input, initial prior
	$P_{t+1|t}=\frac{\partial f_{t|t}}{\partial x}P_{t|t}{\frac{\partial f_{t|t}}{\partial x}}^{T}+W_{t}$	$h_{t}$: relates the observation $z_{t,n}$ and the current state
		$P_{t}$: uncertainty covariance
		$W_{t}$: input noise covariance

**Table 2:** Dataset characteristics.

Real Datasets			
Dataset	Images	Distance	Publicly Available
Dataset 1: *Innova trajectory*	1450	174 m	[43]
Dataset 2: *Bovisa 10-04-2008*	57,733	1310 m	[44]

## Abbreviations

The following abbreviations are used in this manuscript:

CCD	charge-coupled device
EKF	extended Kalman filter
GP	Gaussian process
GPS	global positioning system
KL	Kullback–Leibler divergence
MLE	maximum likelihood estimator
SURF	speeded-up robust features
SVD	singular value decomposition
RMSE	root mean square error
